# The Role of Inflammatory Biomarkers in Predicting Postoperative Fever Following Flexible Ureteroscopy

**DOI:** 10.3390/medicina61081366

**Published:** 2025-07-28

**Authors:** Rasha Ahmed, Omnia Hamdy, Atallah Alatawi, A. Alhowidi, Nael Al-Dahshan, Ahmad Nouraldin Alkadah, Siddique Adnan, Abdullah Mahmoud Alali, Yazeed Hamdan O. Alwabisi, Saleh Alruwaili, Muteb Bandar Binmohaiya, Amany Ahmed Soliman, Mohamed Elbakary

**Affiliations:** 1 Urology Department, Faculty of Medicine for Girls, Al-Azhar University, Cairo 11651, Egypt; amaniahmed.8@azhar.edu.eg; 2 Urology Department, Specialized King Fahd Hospital, Tabuk 31444, Saudi Arabia; aal-atawi@moh.gov.sa (A.A.); aalhowidi@moh.gov.sa (A.A.); naldahshan@moh.gov.sa (N.A.-D.); aalkadah@moh.gov.sa (A.N.A.); sidiqueak@moh.gov.sa (S.A.); aalali33@moh.gov.sa (A.M.A.); yalwabisi@moh.gov.sa (Y.H.O.A.); salruwaili30@moh.gov.sa (S.A.); mbinmohaiya@moh.gov.sa (M.B.B.); melbakary@moh.gov.sa (M.E.); 3 Engineering Applications of Lasers Department, National Institute of Laser Enhanced Sciences, Cairo University, Giza 12613, Egypt; 4 Urology Department, Faculty of Medicine, Tanta University, Tanta 31527, Egypt

**Keywords:** flexible ureteroscopy, neutrophil-to-lymphocyte ratio, systemic immune–inflammation index, platelet-to-lymphocyte ratio

## Abstract

*Background and Objectives*: Flexible ureteroscopic surgery is a common minimally invasive procedure utilized for the management of various urological conditions. While effective, postoperative complications such as fever can occur, necessitating the identification of reliable biomarkers for early detection and management. In this study, we specifically evaluated the predictive performance of three preoperative hematologic indices: the neutrophil-to-lymphocyte ratio (NLR), platelet-to-lymphocyte ratio (PLR), and systemic immune–inflammation index (SII). *Materials and Methods:* By systematically comparing these biomarkers through receiver operating characteristic (ROC) curve analysis and logistic regression modeling, we aimed to identify the most accurate predictor of postoperative fever development. Our cohort included patients who developed postoperative fever, many of whom exhibited normal WBC counts, allowing us to evaluate the discriminatory power of alternative inflammatory biomarkers. *Results*: Among the 150 patients, 32 developed postoperative fever. Conventional WBC counts did not predict fever, with 91% of feverish individuals having normal WBC values. In the ROC curve analysis, NLR outperformed SII (AUC 0.847, cutoff 796) and PLR (AUC 0.743, cutoff 106), with an AUC of 0.996 at 2.96. A combined logistic model achieved 100% sensitivity and 91% specificity (AUC = 0.996). *Conclusions*: This study addresses a critical gap in perioperative monitoring by validating readily available complete blood count-derived ratios as clinically meaningful predictors of postoperative inflammatory responses.

## 1. Introduction

Flexible ureteroscopy (FURS) is a new, minimally invasive surgical method used to treat a variety of urological conditions, such as kidney stones and upper urinary system blockages [1]. Compared to traditional open surgery, this approach provides considerable benefits, such as smaller incisions, lower postoperative discomfort, shorter hospital stays, and faster recovery times, leading to improved patient satisfaction and clinical results [2]. Even with its benefits, postoperative problems are still possible for patients receiving FURS, and one of the most commonly reported adverse events is fever. These issues can cause prolonged hospital stays, hinder rehabilitation, and, in extreme situations, result in systemic infections, highlighting the importance of early discovery and treatment [3]. Because surgical trauma and mechanical manipulation during FURS can activate immunological pathways and cause the release of pro-inflammatory cytokines, the body’s systemic inflammatory response plays a crucial role in postoperative recovery [4]. Although this reaction is a normal component of the healing process, it can occasionally become so severe that it increases the risk of problems. In light of this connection, inflammatory biomarkers such procalcitonin, white blood cell count, and C-reactive protein (CRP) have drawn interest as possible indicators of surgical results. By keeping an eye on these indicators, doctors may be able to spot high-risk patients sooner, facilitating prompt interventions and better recovery rates [5].

The neutrophil-to-lymphocyte ratio (NLR), platelet-to-lymphocyte ratio (PLR), and systemic immune–inflammation index (SII) have become potential hematological indicators that shed light on a patient’s systemic inflammatory condition. These indices, which are based on standard complete blood count (CBC) values, provide an accessible and affordable way to assess the degree of inflammation and immune response [6]. Higher SIIs, NLRs, and PLRs have been linked to worse postoperative outcomes in a number of surgical specialties, including general surgery, cardiovascular surgery, and oncology [7,8]. Their capacity to represent underlying immunological dysregulation, elevated stress reactions, and possible infection risks after surgery gives them prognostic utility. Examining the relationships between these inflammatory markers and postoperative fever has important therapeutic implications for FURS [9]. A frequent but possibly dangerous side effect, postoperative fever can be a sign of an infection, systemic inflammation, or other negative reactions [10]. Clinicians can enhance risk stratification, allowing for earlier problem diagnosis and more individualized surgical therapy, by examining preoperative and postoperative trends in the SII, NLR, and PLR. For example, to reduce fever and other inflammatory sequelae, individuals with consistently elevated NLRs or SIIs following FURS may benefit from closer monitoring, preventative antibiotics, or anti-inflammatory treatments [11]. This strategy is consistent with the increased emphasis on precision medicine in urology, in which biomarker-guided treatments improve patient outcomes while reducing unnecessary interventions.

When studying postoperative complications after flexible ureteroscopic surgery, systemic inflammatory indicators such as the SII, NLR, and PLR have received a lot of attention as prospective biomarkers for predicting outcomes like postoperative fever. Preoperatively monitoring these inflammatory markers can give healthcare personnel significant insights into the patient’s inflammatory status and potential susceptibility to postoperative problems such as fever. Monitoring changes in the SII, NLR, and PLR postoperatively may potentially contribute to the early detection of problems, allowing for timely intervention and better patient outcomes. This study aims to investigate the relationships between elevated inflammatory markers and postoperative fever in patients undergoing flexible ureteroscopic surgery. By analyzing the prevalence of SIIs, NLRs, and PLRs alongside the incidence of postoperative fever, this research seeks to contribute to the identification of potential biomarkers for predicting and managing postoperative complications in urological surgical settings.

## 2. Materials and Methods

The primary outcome of the current study is the development of postoperative fever (body temperature ≥ 38 °C within 48 h after surgery), which is a clinically relevant signal of possible infections or inflammation. The secondary outcomes included (1) the diagnostic performance of inflammatory markers (NLR, PLR, and SII) in predicting postoperative fever, as measured by sensitivity, specificity, and ROC curve analysis, and (2) the relationships between predefined inflammatory marker thresholds and the risk of postoperative complications.

### 2.1. Samples

**Figure 1:**
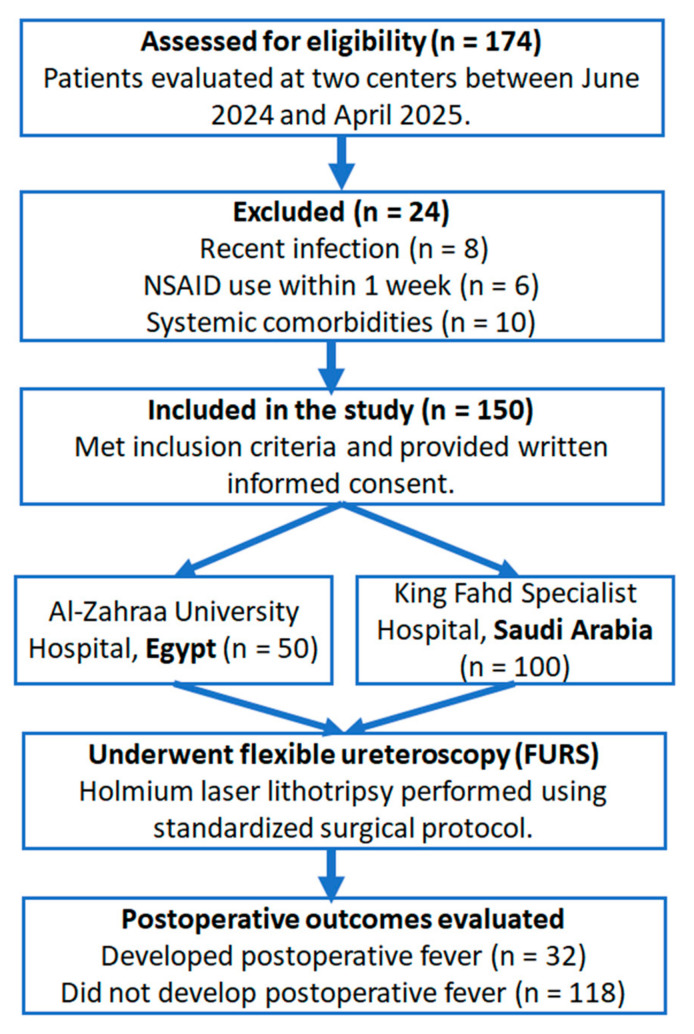
CONSORT-style flowchart of patient enrollment, inclusion, and outcome stratification.

A prospective, observational, two-center cohort study was conducted at the Urology Departments of Al-Zahraa University Hospital (Egypt) and King Fahd Specialized Hospital, Tabuk (Saudi Arabia), between June 2024 and April 2025. As illustrated in Figure 1, the study involved 150 individuals in total (50 patients were enrolled from Al-Zahraa University Hospital and 100 patients from King Fahd Specialist Hospital). Prior to their involvement, each participant gave written informed consent attesting to their voluntary consent to participate in all clinical assessments and surgical procedures in compliance with ethical research guidelines. All patients had a full clinical evaluation, which included detailed personal and medical histories, as well as comprehensive physical and general examinations. Non-contrast computed tomography (CT) of the abdomen and pelvis was used to definitively diagnose renal calculi. Routine laboratory testing included urine, serum creatinine, blood urea nitrogen, liver function tests, complete blood count (CBC), and coagulation profiles to assess the patients’ baseline health state and eligibility for inclusion. Outliers were evaluated using boxplot and histogram visualization. No imputation was performed; all available data were analyzed. This observational study was reported in accordance with the STROBE guidelines (see Supplementary Table S1).

Patients aged 18 and above who had unilateral or bilateral renal stones with a maximum diameter of 2 cm or less were eligible for participation. Although patients were selected using thorough inclusion/exclusion criteria, no randomization or stratified allocation was used. The study design included successive enrollment without allocation or stratification. Accordingly, the current study is classified as a prospective observational cohort study. Exclusion criteria included the presence of renal stones in a calyceal diverticulum, stones larger than 2 cm, multiple renal calculi, or anatomical anomalies such as ureteropelvic junction blockage or ureteral strictures. Patients in poor general health, with systemic comorbidities, a history of drug or alcohol abuse, use of nonsteroidal anti-inflammatory drugs (NSAIDs) within the previous week, and recent infections, such as upper respiratory tract infection within one month or urinary tract infection at the time of assessment, were also excluded. Additionally, patients with sepsis or a known cancer, those who were pregnant, or those whose clinical records were lacking or insufficient for a complete assessment were not included. Despite this, strict exclusion criteria may limit generalizability. They were applied to isolate the effect of preoperative inflammatory markers on postoperative fever, minimizing potential confounding.

All patients were given antibiotic prophylaxis prior to surgery (intravenous ceftriaxone 1 g 30 min before surgery), and retrograde intrarenal surgery (RIRS) was performed under general anesthesia. In all cases, a ureteral access sheath was originally inserted to enable instrumentation. A flexible ureteroscope was then used for stone visualization and treatment. A holmium:YAG laser was used to perform laser lithotripsy. Depending on the clinical setting, a 200 µm laser fiber (maximum power 12 W) or 365 µm laser fiber (maximum power 18–20 W) were used. The “popcorn” and “dusting” techniques were used as fragmentation strategies, with laser settings modified accordingly. These settings were selected to maximize stone fragmentation while reducing ureteral damage.

Blood samples were collected 24 h before flexible ureteroscopy and analyzed with a Sysmex XN 1000 automated hematology analyzer (Sysmex Corporation, Kobe, Japan). Cell numbers are reported as 10^3^/μL. The NLR, obtained by dividing the absolute neutrophil count by the absolute lymphocyte count, and the PLR, derived from the ratio of platelet count to lymphocyte count, are recognized as markers of systemic inflammation and the immune response [12]. The SII, calculated based on peripheral blood counts of platelets, neutrophils, and lymphocytes, serves as a comprehensive indicator of the systemic inflammatory response. Complete blood counts obtained 24 h preceding to surgery and before any indications of postoperative fever were used to compute the NLR, PLR, and SII. Elevated SIIs are associated with a heightened inflammatory state, suggesting a potential link to postoperative complications. Ratios were calculated in MATLAB R2023b utilizing raw CBC outputs, with no rounding before analysis. Antibiotic prophylaxis was given prior to the intervention. Flexible ureteroscopy was performed and lithotripsy was carried out by a holmium: YAG laser (HL).

### 2.2. Descriptive Statistics and Visualization

To characterize the distribution and interrelationships of preoperative inflammatory markers, descriptive statistical analyses were performed on white blood cell counts (WBCs), NLR, PLR, and SII. For each parameter, measures of the central tendency and dispersion, including the mean, median, standard deviation, interquartile range (IQR), and range (minimum–maximum), were computed. Histograms were constructed to assess the distribution patterns and identify skewness or potential outliers within each marker. In addition, a correlation matrix was generated using Pearson’s correlation coefficients to evaluate the linear relationships between the four markers. A scatterplot matrix was also produced to visualize pairwise relationships. All analyses were conducted using MATLAB R2023b, with visualizations created using built-in plotting functions. The statistical analysis was conducted by a multidisciplinary team consisting of clinical urologists and a biomedical data scientist.

### 2.3. Receiver Operating Characteristic (ROC) Curve

The ROC (receiver operating characteristic) curve is a graphical tool used to assess the accuracy of diagnostic tests by plotting sensitivity against 1-specificity [13,14]. Sensitivity represents the proportion of true positives, while 1-specificity corresponds to the false positive rate. The area under the ROC curve (AUC) is a key measure of a test’s effectiveness: the larger the AUC, the more accurate the test is [15,16]. The ROC curve is constructed using values derived from true positives, true negatives, false positives, and false negatives [17]. On the graph, sensitivity is displayed on the Y-axis, and 1-specificity (false positive rate) is plotted on the X-axis. In this study, the ROC curves were produced using the MATLAB R2023b software framework, utilizing a custom MATLAB function. ROC curves were created using MATLAB’s perfcurve function. This function generates the ROC curve and computes its associated parameters by plotting 1-specificity against sensitivity for two data categories (pre- and post-surgery). Bootstrapped confidence intervals (95% CI) for the AUC were computed using 1000 bootstrap iterations in order to evaluate the robustness of diagnostic performance. For each biomarker (NLR, PLR, and SII), receiver operating characteristic (ROC) curves were generated, and the Youden index was utilized to determine the best cut-off values.

## 3. Results

Our investigation involved 150 patients. The median age was 40 years. The most common symptom at the time of presentation was pain. The average diameter of the stones was (1.3 ± 0.4), with the renal pelvis being the most common location.

### 3.1. Descriptive Findings and Correlations

**Figure 2:**
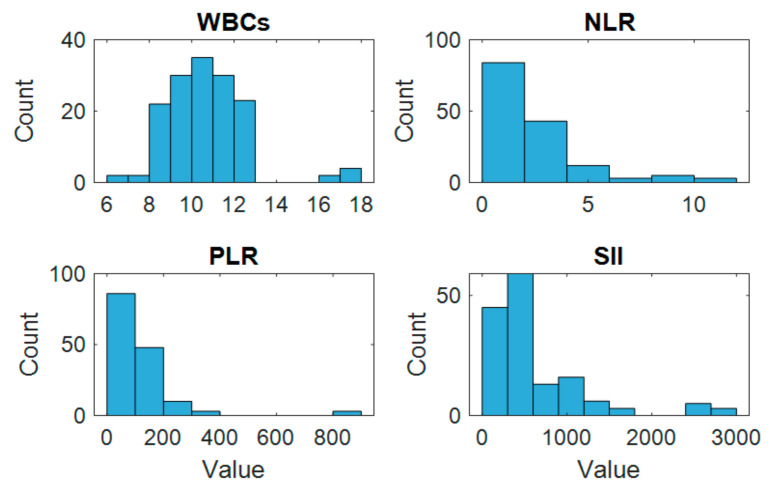
Histograms showing the distribution of preoperative inflammatory biomarkers (WBCs, NLR, PLR, and SII). WBCs and PLRs were nearly symmetrically distributed, whereas NLRs and SIIs were right-skewed, indicating a subset of patients with increased inflammatory responses. Several outliers were observed, particularly in the SII panel, indicating clinical variability.

The histogram analysis revealed varying distribution patterns among the preoperative inflammatory markers, as illustrated in Figure 2. WBCs and PLRs showed relatively symmetric distributions, while NLRs and SIIs exhibited right-skewed patterns, indicating the presence of higher values in a subset of patients. Several markers showed visible outliers, particularly within the SII distribution.

**Figure 3:**
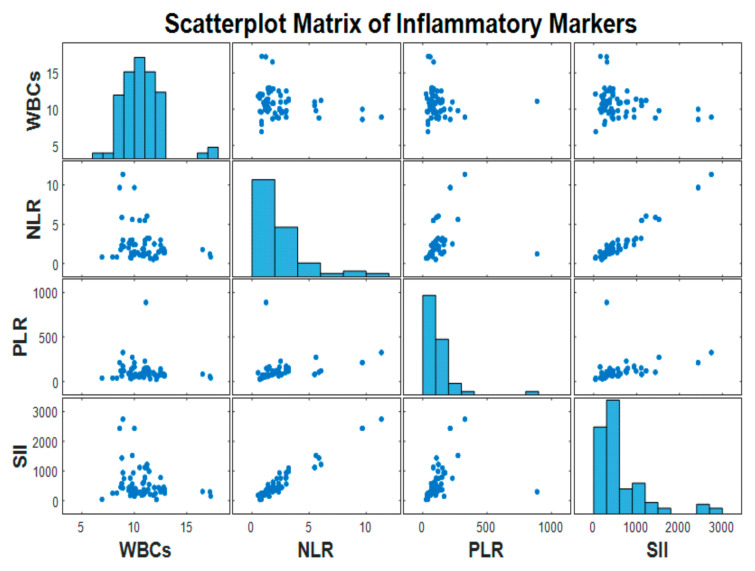
WBC scatterplot matrix and correlation analysis with the NLR, PLR, and SII. The NLR and SII showed a substantial positive correlation (r = 0.97). The PLR exhibited moderate correlations with both the NLR and SII, whereas WBCs had weak or inverse interactions with other markers. The results presented back up the integrative significance of ratio-based indices in detecting systemic inflammation.

The correlation matrix demonstrated a range of positive associations among the markers. Notably, a strong positive correlation was observed between the NLR and SII, suggesting that as the NLR increases, the SII also tends to rise. Moderate correlations were also seen between the PLR and both the SII and NLR. These findings suggest that although the markers are related, each may reflect different aspects of the systemic inflammatory response. The scatterplot matrix (Figure 3) further illustrated these linear trends and supported the presence of collinearity among some variables, particularly between the NLR and SII.

The correlation analysis revealed notable interrelationships among the preoperative inflammatory markers. A strong positive correlation was observed between NLR and SII (r = 0.97), indicating that these two markers closely track each other and likely reflect similar aspects of the systemic inflammatory response. Moderate positive correlations were also found between the PLR and SII (r = 0.30) and between the PLR and NLR (r = 0.27), suggesting a partial overlap in their inflammatory profiles. In contrast, WBCs showed weak and negative correlations with the other markers—most notably with the SII (r = –0.28) and NLR (r = –0.27)—indicating that the WBC count may not consistently vary in parallel with these ratio-based indices. These findings support the notion that composite indices such as the NLR, PLR, and especially SII may provide more sensitive and integrative reflections of systemic inflammation compared to WBC counts alone.

Among the studied group, a substantial proportion, specifically 21% of the patients (32 participants), reported experiencing postoperative fever after undergoing flexible ureteroscopic surgery, even though 91% of them had normal white blood cell (WBC) counts. This shows that standard WBC monitoring is inadequate as a predictor of this frequent postoperative complication. This striking observation prompted us to investigate more sensitive inflammatory biomarkers that could reliably anticipate febrile responses following surgery. This subgroup study draws attention to the possible diagnostic drawbacks of using the total WBC count alone to predict postoperative fever after flexible ureteroscopy. The WBC count is a common indicator of inflammation, but in 91% of cases, it was unable to identify patients who were feverish even if they had normal WBC counts after surgery. The power analysis was based on the observed AUC for the NLR (0.996), a two-tailed alpha of 0.05, and an expected fever rate of 21%. Using MedCalc^®^ (v22.014) software and typical ROC curve assumptions, we determined that at least 24 participants per group would be necessary for achieving 80% power. Our actual sample (*n* = 150; 32 febrile participants, 118 non-febrile participants) exceeds the criteria.

**Figure 4:**
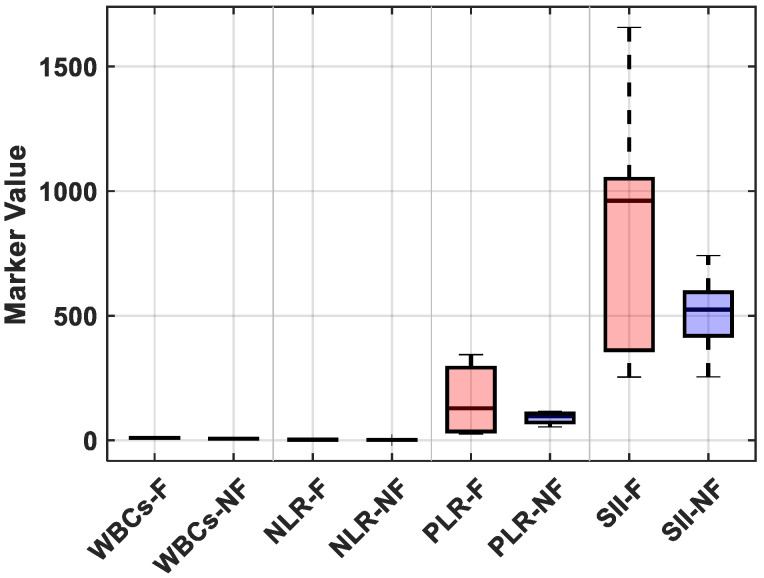
A boxplot comparing preoperative inflammatory markers (WBCs, NLR, PLR, and SII) between individuals who developed postoperative fever and those who did not after flexible ureteroscopy. The figure depicts larger distributions of the NLR and SII in febrile individuals, although WBCs exhibited significant overlap between the two groups. Each box represents the interquartile range, with the median shown by a horizontal line; whiskers denote data within 1.5 times the IQR; and outliers are displayed as individual points.

Postoperative fever was defined in our study as a body temperature ≥ 38 °C within 48 h following FURS. Of the 32 patients who experienced fever, 25% showed signs of an early systemic inflammatory response (SIRS) and received escalation of care, which included intravenous broad-spectrum antibiotics and extended hospital observation. No patients developed sepsis or needed to be admitted to the intensive care unit (ICU), most likely because of the early intervention based on fever detection and predetermined antibiotic protocols. Postoperative fever, in most cases, triggered additional laboratory tests, including a complete blood count, serum procalcitonin levels, and urinalysis with culture. Therefore, even though the fever was temporary in the majority of patients, it increased the use of diagnostic and therapeutic resources, highlighting the significance of accurate risk stratification to guide preemptive management. The distributions of each marker were compared between patients who were feverish and those who were not, further visualizing these findings. The febrile group had significantly higher median values for the NLR and SII, as shown in Figure 4; however, there was a notable overlap in WBC counts between the groups (see Supplementary Table S2 for detailed descriptive statistics).

### 3.2. ROC Curve Analysis

**Figure 5:**
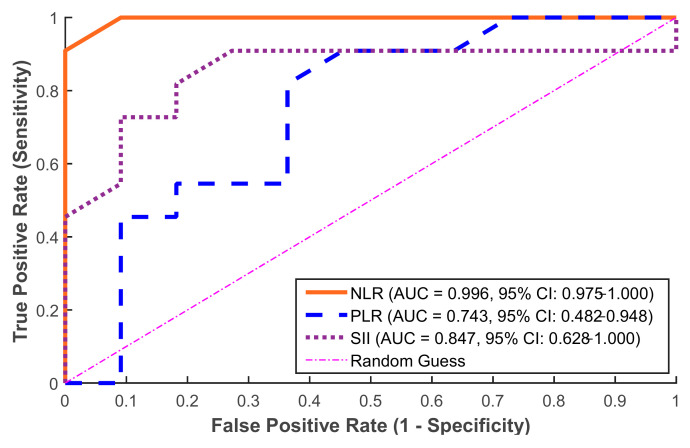
ROC curves for preoperative markers predicting postoperative fever. The NLR achieved the highest performance (AUC = 0.996, 95% CI: 0.975–1.000), followed by the SII (AUC = 0.847, 95% CI: 0.628–1.000) and PLR (AUC = 0.743, 95% CI: 0.482–0.948). Cut-off values were selected using the Youden index.

The obtained ROC curve results showed that inflammatory markers had a remarkable ability to discriminate in the prediction of postoperative fever, as illustrated in Figure 5. Strong predictive value was established by the ROC curve analysis for the NLR (AUC = 0.996, 95% CI: 0.975–1.000), SII (AUC = 0.847, 95% CI: 0.628–1.000), and PLR (AUC = 0.743, 95% CI: 0.482–0.948). To ensure internal validation, these values were verified using bootstrap resampling (1000 iterations). According to these results, the NLR is the most reliable standalone predictor in this group followed by the SII, which also provides a strong predictive accuracy. On the other hand, the PLR performed more moderately with AUC = 0.743. Moreover, the optimal thresholds for clinical prediction (i.e., cut-off value) for the three markers were calculated (see Table 1).

**Figure 6:**
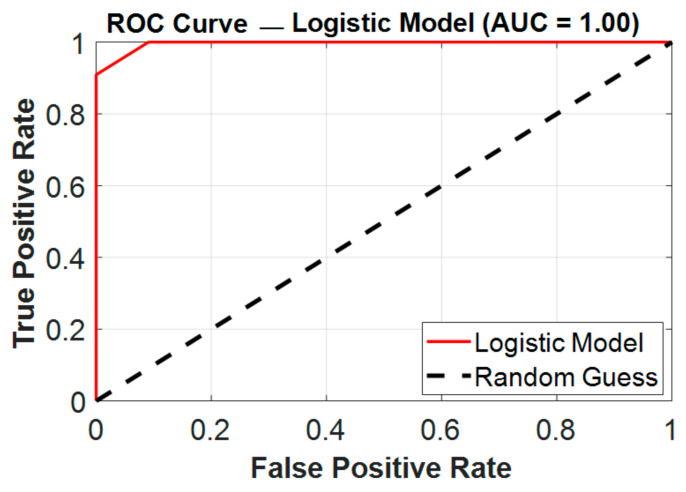
ROC curve for the combined logistic regression model based on all 3 markers.

In this cohort, the NLR showed excellent discrimination (AUC = 0.996) when used alone. However, in order to assess possible synergistic effects and improve the prediction accuracy, we also examined and compared the combined model including the NLR, SII, and PLR. Through their different pathophysiological pathways, these three biomarkers combined to achieve perfect classification (AUC = 1.000), as presented in Figure 6, which indicates complementary value: the PLR offers additional immunological context, SII represents platelet-mediated immune responses, and NLR captures acute neutrophilic inflammation.

With a sensitivity of 100% as opposed to 91% for the NLR, this multi-parametric method proved very useful in removing false negatives that can have clinical repercussions in postoperative monitoring. Although the slight increase in the AUC (from 0.996 to 1.00) may seem insignificant on a numerical level, it signifies a significant gain in predicting reliability, particularly when taking into account possible differences in actual clinical populations where single-marker performance may vary. When avoiding missing high-risk patients is the primary objective, this trade-off could be therapeutically helpful, particularly when the consequences of delayed infection control are severe. Furthermore, the combined model might be used as a foundation for additional validation in more variable cohorts where the NLR’s independent performance might fall short of near-perfect values. Additionally, the combined model is more robust to individual patient variability than any single parameter alone, most likely due to its capacity to account for many elements of the inflammatory cascade. These results imply that although the NLR is still considered an excellent first-line screening method because of its ease of use and exceptional performance, the combined model provides a more thorough evaluation for crucial clinical decision-making situations when optimizing predictive accuracy is crucial. Future research should examine whether the enhanced performance of this multi-marker strategy is maintained across a range of surgical populations and healthcare environments.

These presented results imply that WBCs alone may not be a sensitive enough signal to predict postoperative fever, as a significant percentage of febrile cases occurred even when WBC counts were normal. The preoperative NLR, on the other hand, was a more reliable indicator. These findings add credence to the increasing amount of data highlighting the NLR’s exceptional sensitivity as a biomarker of early systemic inflammation or subclinical inflammation, which may not yet show up as leukocytosis. Therefore, even when conventional measures like the WBC count are within normal ranges, adding the NLR to preoperative risk stratification techniques may improve the early identification of patients at higher risk for postoperative infection problems. These results underscore the potential significance of the SII, NLR, and PLR as predictive biomarkers for identifying patients at a higher risk of developing postoperative complications, particularly fever, following flexible ureteroscopic surgery. The high prevalence of increased inflammatory marker levels alongside the notable incidence of postoperative fever highlights the relevance of monitoring these indices as part of preoperative assessments and postoperative care strategies to enhance patient management and outcomes in this clinical setting.

## 4. Discussion

Renal stone care has typically included action for symptomatic cases, with observation reserved for asymptomatic stones. However, some asymptomatic individuals may still require intervention [18]. The introduction of RIRS has increased therapeutic choices by providing effective access to intrarenal calculi at a lower risk than percutaneous nephrolithotomy (PNL), avoiding consequences such as hemorrhage, visceral damage, and urine leakage [19]. The use of a ureteral access sheath allows for flexible ureteroscopy (fURS), whereas holmium laser lithotripsy allows for effective stone fragmentation, typically allowing for same-day treatments. High stone-free rates and low retreatment rates have made fURS a potentially superior option to extracorporeal shock wave lithotripsy (ESWL) [20,21].

All patients, including those with sterile urine cultures, received prophylactic antibiotics prior to surgery, in accordance with European Association of Urology standards [22]. Given the link between longer operating periods and postoperative infection complications, most likely due to prolonged exposure to irrigation fluids, procedures lasting more than 90 min (measured from sheath insertion) were conducted in stages, as needed [23]. Postoperative infections can range in severity from transitory bacteremia to sepsis, emphasizing the significance of discovering accurate prognostic biomarkers. Neutrophilia and lymphopenia are recognized to contribute to an inflammatory environment conducive to nephrolithiasis, but their function in the postoperative infection risk is still being investigated. Inflammatory indices such as the NLR and PLR have shown potential in predicting systemic inflammatory response syndrome (SIRS) after endourological treatments, including ureteroscopy and PNL [24].

An integrated indicator of the systemic inflammatory status, the systemic immune–inflammation index (SII), was introduced by Hu et al. [25] and includes the platelet count in addition to neutrophil and lymphocyte counts. As a stand-alone indicator of neuroendocrine stress and immune–inflammatory activity, Zahorec et al. [26] suggested the neutrophil-to-lymphocyte ratio (NLR) as a useful and sensitive metric. This ratio illustrates the dynamic interaction between lymphocytes, which are essential for adaptive immunity, and neutrophils, which stand for the innate immune response. The NLR, which may be produced by dividing the absolute neutrophil count by the absolute lymphocyte count from a standard complete blood count, has become a widely available measure of systemic inflammation. The severity of sepsis and other systemic infections, such as bacteremia, may now be evaluated using the NLR, which was first identified as a marker for SIRS in critically ill patients.

From a biological perspective, increased NLRs and SIIs indicate an underlying inflammatory shift marked by lymphopenia and neutrophilia, two indicators of acute systemic stress and immunological dysregulation. In addition to being powerful producers of cytokines, reactive oxygen species, and proteolytic enzymes, neutrophils aid in the early stages of innate immune responses. Simultaneous suppression of lymphocytes may indicate compromised adaptive immunity, resulting in a pro-inflammatory environment that increases the risk of febrile complications after surgery. The predictive ability of these biomarker patterns in our investigation may be explained by their alignment with well-established theories of immunological activation brought on by surgery.

By examining the prevalence of SII, NLR, and PLR levels as well as the incidence of postoperative fever, this study aims to contribute to the development of possible biomarkers for predicting and controlling postoperative complications in urological surgical settings. Numerous studies have examined the NLR reference levels in populations of healthy adults. One of the biggest studies was carried out in a cohort of 9427 people from New York State by Azab et al. [27], who reported a mean NLR of 2.15 with a reference range of 1.71–2.28. There were racial disparities, with African Americans displaying the lowest average NLR (1.76), followed by Caucasians (2.24), and Hispanics (2.08). Smoking, obesity, and diabetes mellitus were among the major risk factors associated with mild increases in the NLR. The results of other trials conducted on healthy people were comparable. The mean NLR was 1.95 (range 0.77–4.5) in a sample of 236 young people from Indonesia, but Forget et al. [28] found that the mean NLR in a Belgian population of 413 healthy adults was 1.65. In the context of acute care, Ljungström et al. [29] prospectively assessed 1572 persons with suspected sepsis and discovered that the NLR was a superior inflammatory marker to C-reactive protein (CRP), but less accurate than procalcitonin for diagnostic purposes. Sedat Yahşi et al. [30] found that preoperative SIIs, NLRs, and PLRs were considerably higher in individuals who developed systemic inflammatory response syndrome (SIRS) after ureteroscopy. However, their ROC curve analysis found the NLR as the only independent predictor of postoperative SIRS, with a recommended limit of the NLR > 2.98 resulting in 73.3% sensitivity and 71.3% specificity.

In our investigation, the ROC curve analysis revealed that preoperative inflammatory indicators had a high predictive value for identifying individuals at risk of developing postoperative fever after flexible ureteroscopy (FURS), as shown in Figure 6. Among the examined indicators, the NLR demonstrated complete discrimination, making it the most reliable solo predictor in our study. These results are comparable with those of [30], despite changes in the surgical method, highlighting the predictive value of the NLR in endourological settings. The SII also had a high predictive capacity, indicating its potential as an additional marker. In contrast, the PLR performed more moderately, putting it behind the NLR and SII in diagnostic accuracy. The individual marker distribution analysis provided additional support for these findings. Despite being a commonly used infection measure, the WBC count did not consistently predict postoperative fever. Notably, several febrile patients had WBC counts within normal ranges, indicating low sensitivity. In contrast, NLRs had a broader and more informative range, demonstrating their superiority as a prognostic marker. Similarly, the SII showed a wide range and correlated positively with febrile outcomes, albeit to a smaller extent than the NLR. The PLR, with a more variable and right-skewed distribution, was the least informative of the three indices. These results justify the incorporation of the NLR, and to a lesser extent the SII, into preoperative risk classification algorithms, even when conventional measurements like WBC counts are within normal ranges.

Recent research confirms the significance of biomarkers such NLR, IL-6, CRP, procalcitonin, and lactate levels in diagnosing and monitoring sepsis, systemic infection, and SIRS [31]. For validation of the results, we compared our findings with prior research, particularly the study by Sedat Yahşi et al. [30], which reported an NLR cutoff of 2.98 with an AUC of 0.73 for predicting systemic inflammatory response syndrome (SIRS) following ureteroscopy. Despite the similar threshold (2.96) found in our study, our cohort’s greater AUC might be explained by variations in surgical procedures (FURS vs. semirigid URS), sample features, or exclusion criteria that minimized clinical heterogeneity. These elements probably improved our model’s signal-to-noise ratio; therefore, they should be taken into account when extrapolating these results. Moreover, our results are consistent with those of Yang et al. [32], who retrospectively examined 405 patients undergoing percutaneous nephrolithotomy (PCNL) and observed that an elevated preoperative NLR was significantly associated with the development of febrile urinary tract infection (fUTI), with an odds ratio of 1.49 and an AUC of 0.718. They reported a cut-off value of 2.71, which is closely comparable to our obtained threshold of 2.96 for predicting postoperative fever following FURS. Although their study focused on PCNL, a more invasive operation, their findings support the use of the NLR as a simple and effective predictor for infection risk in endourologic surgery. Notably, Yang et al. proved the robustness of the NLR using both univariate and multivariate logistic regression models, confirming its predictive efficacy across many statistical techniques. In comparison, our study has an even greater discriminative performance, which could be attributed to changes in procedural invasiveness, patient selection, or our prospective data collection methodology.

Our results have also been validated in non-urologic settings, such as autoimmune illnesses. Wu et al. [33] showed considerably higher levels of the NLR and PLR in 116 untreated patients with systemic lupus erythematosus (SLE) compared to healthy controls. Both indicators showed a positive correlation with disease activity scores, and the study determined NLR and PLR cut-off values of 2.26 and 203.85, respectively, for predicting severe SLE activity. These findings are significant because they confirm the biological plausibility and diagnostic adaptability of the NLR and PLR across a variety of inflammatory diseases. Our analysis confirms these results in a surgical context. SLE and postoperative fever have different etiologies, although both involve acute or chronic systemic inflammation. The continuous rise of the NLR and PLR in inflammatory situations such as infection and autoimmunity lends confidence to their roles as robust, non-specific markers of immune system activation. Furthermore, Wu et al.’s reported NLR cutoff of 2.26 for high disease activity is similar to our fever-predictive threshold of 2.96, indicating the indices’ translational validity.

As an appropriate screening tool, a proposed decision threshold of an NLR > 2.96 may be used. This could improve early risk categorization when included in standard preoperative CBC assessments. Additional validation is necessary to confirm cost-effectiveness and utility in larger, diverse cohorts. Despite the fact that the NLR alone showed remarkable discriminative ability for predicting postoperative fever, our logistic regression model that the combined NLR, SII, and PLR provided a comparably high but marginally lower AUC. Crucially, the amalgamated model attained 100% sensitivity with a slightly diminished specificity of 91%, indicating a possible therapeutic benefit in situations when minimizing false positives is not as important as the early detection of infection complications. This small compromise could have therapeutic benefits. Early management of surgical patients who are at a higher risk of developing postoperative fever may be able to stop the development of more severe consequences like sepsis. The SII and PLR may increase model resilience in bigger or more diverse populations, even though they may not enhance overall discrimination much in this dataset. Consequently, the combined model may be useful in risk-averse therapeutic practices where maximizing sensitivity is the aim, even though the NLR is still the best predictor when used alone.

### Limitations

The present study has several limitations, which should be acknowledged. First, the sample size of 150 patients, while sufficient to detect relevant trends, was not determined using a formal sample size calculation because the study was designed as an exploratory observational analysis. As a result, the statistical ability to detect subtle relationships or subgroup differences may be restricted, and thus future research should use a power analysis to guide enrollment for desired outcomes. However, we addressed this by conducting a post hoc power analysis, which verified that our sample was sufficient to detect statistically significant variations in biomarker performance, particularly for the NLR. Second, the number of patients who had postoperative fever (*n* = 32) was limited, which may have elevated the performance metrics (e.g., AUC) and increased the risk of overfitting, particularly for the NLR marker. We addressed this risk by using bootstrapped confidence intervals and highlighting the importance of external validation. Third, this study used a single preoperative measurement of inflammatory markers and did not include serial measurements or long-term outcomes, limiting insights into the temporal dynamics of the indicators.

Moreover, the distribution of enrolled patients was not balanced (50 from Egypt and 100 from Saudi Arabia), and institutional-level differences were not examined independently, despite the fact that the trial was carried out across two centers. Even though the surgical and laboratory procedures were the same at both centers, the results might have been affected by unmeasured variability. Our restricted inclusion and exclusion criteria, such as excluding individuals with recent infections, systemic illnesses, immunosuppression, or NSAID usage, were intended to minimize confounding and clinical noise. However, because of this selected methodology, our findings are limited in their applicability to the larger population undergoing flexible ureteroscopy. These exclusions, which focus on reasonably healthy patients, may also introduce some selection bias.

Beyond bootstrapping, no internal validation methods (such as k-fold cross-validation) were used, and the study was not prospectively registered. In order to further improve predictive modelling techniques, we recommend future research to include prospective registration, multicenter harmonization, and blinded outcome adjudication. Finally, other validated sepsis-related biomarkers (e.g., procalcitonin and IL-6) were not evaluated, despite the fact that the inflammatory markers employed in this investigation are easily available and generated from standard blood testing. These biomarkers may provide complementary relevance in future research. Despite these limitations, this work offers new evidence in favor of the preoperative use of the NLR and SII as useful and cost-effective measures for postoperative fever risk classification after FURS.

Finally, while this study followed a centrally approved ethical protocol, we admit that one participating center (King Fahd Specialist Hospital) did not provide a separate IRB permission document. However, no site-specific interventions or deviations occurred, and all subjects were recruited using the same consent and procedural standards.

## 5. Conclusions

This study highlights the potential use of preoperative inflammatory biomarkers, especially the NLR and SII, as practical, cost-effective methods for predicting postoperative fever in patients undergoing flexible ureteroscopy. These biomarkers, generated from regular blood testing, may provide clinicians with an early warning signal of increased infection risk, even in the absence of overt leukocytosis. Our findings support the use of the NLR-based risk classification in preoperative assessment regimens. However, given the small sample size and restricted inclusion criteria, more validation in larger and more diverse populations is required. Future research should consider combining these markers with other clinical measures or molecular indications to improve the prediction accuracy. If verified, these biomarkers could help with more personalized perioperative care, increased patient safety, and lower postoperative morbidity in endourology practice.

## Figures and Tables

**Table 1:** The obtained ROC curve parameters.

Marker	Sensitivity	Specificity	AUC	Best Threshold
NLR	91%	100%	0.996	2.96
PLR	82%	64%	0.743	106
SII	82%	82%	0.847	796
Combined	100%	91%	1.000	--

## Data Availability

The datasets used and/or analyzed during the current study are available from the corresponding author upon reasonable request.

## Supplementary Materials

The following supporting information can be downloaded at: https://www.mdpi.com/article/10.3390/medicina61081366/s1, Table S1: STROBE Statement; Table S2: Descriptive Findings and Correlation.
