# Supplementary material for: The Role of Inflammatory Biomarkers in Predicting Postoperative Fever Following Flexible Ureteroscopy
Source: Medicina (Kaunas). 2025 Jul 28;61(8):1366. doi: 10.3390/medicina61081366 (PMC12387940; doi:10.3390/medicina61081366)
Supplement: Supplementary file 1 [file medicina-61-01366-s001.zip › medicina-3712114-supplementary.pdf]

Table S1. STROBE Statement— Checklist of items that should be included in observational cohort studies

Checklist Item	Manuscript Location/Status
**Title and abstract**	Study design clarified as observational cohort in title and abstract.
**Background/rationale**	Introduction provides context on FURS complications and inflammation biomarkers (Section 1).
**Objectives**	Study objective stated: to evaluate NLR, PLR, and SII as predictors of postoperative fever (End of Section 1).
**Study design**	Clearly stated as a multicenter observational cohort study in Methods (Section 2.1).
**Setting**	Conducted at Al-Zahraa University Hospital (Egypt) and King Fahd Hospital (Saudi Arabia) from June 2024–April 2025.
**Participants**	Inclusion: adults ≥18 with ≤2cm renal stones. Exclusion: stones >2cm, diverticulum, sepsis, systemic disease, pregnancy (Section 2.1).
**Variables**	Key variables include WBC, NLR, PLR, and SII; outcome is postoperative fever (Methods and Results).
**Data sources/measurement**	CBC-derived indices from routine labs; methods described in Section 2.2.
**Bias**	Selection bias minimized through strict inclusion/exclusion; no external validation performed.
**Study size**	150 patients included; no formal power calculation reported.
**Quantitative variables**	Handled with descriptive stats, ROC, logistic regression (Section 2.2–2.3).
**Statistical methods**	ROC, AUC, sensitivity, specificity, logistic regression described (Section 2.3).
**Participants (Results)**	150 patients total; flowchart and demographics to be added in revised version (Results section).
**Descriptive data**	Mean, median, SD, IQR, and range reported for all biomarkers (Section 3.1).
**Outcome data**	Postoperative fever incidence (21%) reported; WBC normal in 91% of febrile cases.
**Main results**	NLR AUC=1.00, SII AUC=0.85, PLR AUC=0.74; Combined model AUC=0.996 (Figure 5, Table 1).
**Other analyses**	Combined model using logistic regression tested (Section 3.2).
**Key results**	NLR superior as predictor; WBC poor predictor (Discussion and Conclusions).
**Limitations**	Acknowledged lack of external validation, overfitting, and small febrile group (Discussion).
**Interpretation**	Balanced summary in Discussion highlighting clinical relevance and caution in interpretation.
**Generalizability**	Multi-center design improves applicability, but external validation recommended.
**Funding**	No external funding received (Declarations section).

Table S2. DescripWive Findings and CorrelaWion

Marker	Mean	Median	Standard Deviation (SD)	Interquartile Range (IQR)	Minimum	Maximum
**WBCs**	10.73	10.7	1.82	1.8	6.91	17.27
**NLR**	2.58	1.8	2.31	1.73	0.53	11.33
**PLR**	120.37	83.92	125.47	70.7	25.3	888.1
**SII**	620.45	423.38	576.69	497	41	2743.7

Correlation Matrix:

	**WBCs**	**NLR**	**PLR**	**SII**
**WBCs**	1	−0.26994	−0.09782	−0.27893
**NLR**	−0.26994	1	0.27013	0.97177
**PLR**	−0.09782	0.27013	1	0.29843
**SII**	−0.27893	0.97177	0.29843	1
